# Data of fracture effort in the deformation of the cutting surface of scalpel blades

**DOI:** 10.1016/j.dib.2018.11.141

**Published:** 2018-12-04

**Authors:** Antonio Díaz-Caballero, Arnulfo Tarón-Dunoyer, Roes Hernandez-Ligardo

**Affiliations:** a Department of Oral medicine, Gitouc Research group, Faculty of Dentistry, University of Cartagena, Cartagena, Colombia; b Department of Food Science, Gibae Research group, Faculty of Engineering, University of Cartagena, Cartagena, Colombia

## Abstract

This article contains data related to the research article titled “Deformation of scalpel blades after incision of gingival tissue in pig mandibles. An ex vivo study” (Díaz et al., 2017) [1]. The presented data give information on the Rheological properties of a number 3 Bard Parker scalpel blades from the Paramount® and Elite® brands. The loss of the cutting capacity of the scalpel blades was evaluated, determining fracture efforts by incisions on gingival tissues. The Elite® brand presented greater efforts (41.40; 52.70; 59.00; 61.17; 64.00; 66.78; 72.15 and 74.18 Newton (N)) than the scalpel blades from the Paramount® brand (49.60; 51.40; 51.90; 52.33; 58.96; 62.24 and 69.08 N). The cutting effort increases with the number of cuts performed by each scalpel blade.

**Specifications table**

**Table:** 

**Subject area**	Dentistry, Materials Science.
**More specific subject area**	Rheology, Resistance of materials
**Type of data**	Tables and figure
**How data were acquired**	The fracture stress data on the scalpel blades were obtained using a Shimadzu EZ-S Texture Analyzer. The photographs were taken with a Nikon d7000 camera and a Stereomicroscope of D & D brand Implements, digital to 4.5 magnifications.
**Data format**	Observations, analyzed.
**Experimental factors**	Comparative-descriptive study. Commercial use pig mandibles were used. Cuts with 20 scalpel blades per brand were performed. A texture analyzer with force to perform these cuts was used. A stereo-microscope was used to photograph and compare the surface of the scalpel blades before and after being used.
**Experimental features**	The published data are used to determine the deformation and loss of cutting capacity of the scalpel blades when making incisions on gingival tissues.
**Data source location**	Faculty of Dentistry. University of Cartagena. Cartagena. Colombia.
**Data accessibility**	Data are available in this article
**Related research article**	Díaz, C.A., Tarón, D.A., Hernández, L.R., Camacho, V.A., Fortich, M.N. Deformation of scalpel blades after incision of gingival tissue in pig mandibles. An ex vivo study. Revista Odontológica Mexicana. 21 (3), 2017, pp. 173–179.
	https://doi.org/10.12988/ces.2018.84180www.sciencedirect.com/science/article/pii/S1870199X17300563

**Value of the data**

• Data on fracture efforts at cutting can be used to determine the maximum number of incisions (cuts) that should be performed with the same scalpel blade in a surgical procedure.
• The experimentation model can be replicated for resistance studies of materials and instrumental used in the dental area.
• Data on fracture efforts allow to make comparisons in terms of quality (deformation resistance) of scalpel blades.

## 1 Data

**Fig. 1:**
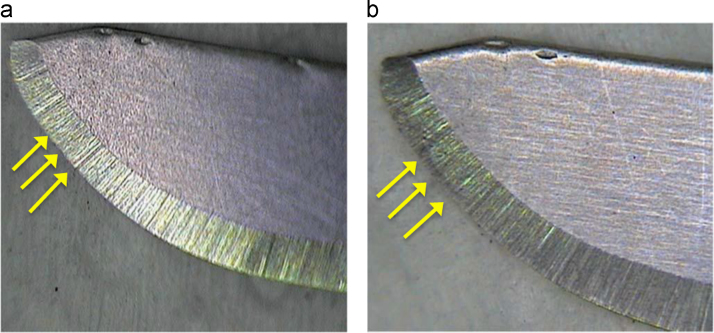
(a) Elite® scalpel blade before being used with an active edge without opacities or deformations. (b) Elite® scalpel blade after being used. On the active edge the opacity of the edge is noted, which translates as a physical deformation of the blade.

**Fig. 2:**
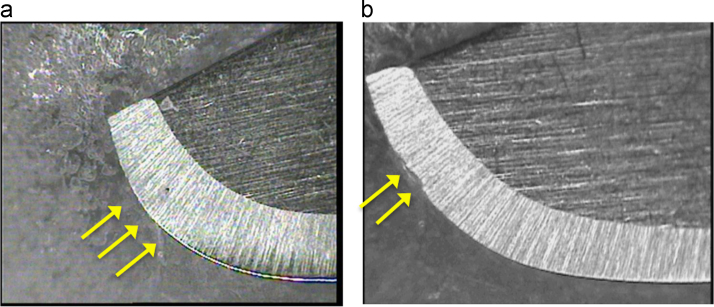
(a) Paramount® scalpel blade before being used with an active edge without opacities or deformations. (b) Paramount® scalpel blade after being used. On the active edge the opacity of the edge is noted, which translates as a physical deformation of the blade.

In this data article it was determined experimentally the values of fracture efforts (cut) of gingival tissues when performing from one to eight successive incisions with number 3 Bard Parker scalpel blades from the Paramount® and Elite® brands. In the Tables 1 and 2, data on fracture efforts from the samples can be observed. The loss of cutting capacity of the scalpel blades from the Paramount® and Elite® brands are related to the number of incisions and the fracture efforts (Newton) when performing between one to four cuts on the tissue. On Figs. 1 and 2 the images of the scalpel blades can be observed before and after performing the cut. Tables 3 and 4 show the variance analysis data of the results.

**Table 1:** Experimental data of the values of fracture efforts (to the cut) of the number 3 Bard Parker scalpel blades from the Paramount® brand.

*Fracture efforts to the cut (Newton)*								
*Experience*	*Number of incisions in the gingival tissue*							
	1	2	3	4	5	6	7	8
1	49.60	51.40	51.90	52.30	58.96	65.24	69.00	71.38
2	49.60	51.34	52.00	52.30	58.90	65.30	69.01	71.38
3	48.99	51.30	52.10	52.28	58.90	65.30	69.00	71.38
4	48.76	51.40	51.90	52.28	58.92	65.31	69.00	71.39
5	49.60	51.25	51.90	52.28	58.96	65.20	69.03	71.38
6	49.88	51.33	51.50	52.20	58.96	65.24	69.01	71.29
7	47.90	51.39	51.80	52.30	58.96	65.30	69.00	71.38
8	48.90	51.00	51.90	52.30	58.95	65.24	69.00	71.39
9	49.60	51.05	51.90	52.28	58.96	65.24	69.15	71.40
10	49.40	51.40	51.90	52.28	58.95	65.23	69.02	71.40
11	49.40	51.22	51.86	52.30	58.92	65.24	69.02	71.39
12	48.90	51.36	51.84	52.30	58.93	65.25	69.00	71.38
13	49.50	51.40	51.00	52.30	58.96	65.25	69.00	71.38
14	49.80	51.40	52.00	52.28	58.96	65.24	69.00	71.38
15	49.60	51.40	51.90	52.28	58.96	65.24	69.01	71.38
16	49.60	51.37	51.90	52.30	58.94	65.24	69.00	71.37
17	49.65	51.38	51.90	52.29	58.94	65.36	69.00	71.39
18	49.64	51.38	51.89	52.29	58.94	65.30	60.01	71.38
19	49.60	51.40	51.89	52.29	58.96	65.30	69.01	71.40
20	49.60	51.41	51.90	52.30	58.96	65.24	69.01	71.37

**Table 2:** Experimental data of the values of fracture efforts (to the cut) of the number 3 Bard Parker scalpel blades from the Elite® brand.

*Fracture efforts to the cut (Newton)*								
Experience	*Number of incisions in the gingival tissue*							
	1	2	3	4	5	6	7	8
1	41.40	52.70	59.00	61.17	64.00	66.78	72.15	74.18
2	41.40	52.68	59.00	61.20	64.00	66.76	72.15	74.17
3	41.41	52.69	59.00	61.22	64.10	66.76	72.13	74.18
4	41.40	52.60	59.00	61.29	64.10	66.78	72.15	74.17
5	41.40	52.70	59.00	61.17	64.10	66.76	72.13	74.17
6	41.35	52.69	58.98	61.17	64.00	66.69	72.14	74.18
7	41.39	52.69	58.91	61.22	64.00	66.80	72.13	74.22
8	41.39	52.70	58.91	61.22	64.00	66.79	72.15	74.17
9	41.39	52.70	58.92	61.19	64.00	66.79	72.15	74.18
10	41.30	52.68	58.91	61.22	64.18	66.78	72.15	74.18
11	41.40	52.68	59.00	61.17	64.00	66.78	72.18	74.16
12	41.40	52.69	58.98	61.17	64.10	66.60	72.17	74.10
13	41.38	52.69	58.96	61.18	64.08	66.78	72.15	74.17
14	41.38	52.70	58.96	61.19	64.08	66.78	72.16	74.17
15	41.39	52.70	59.00	61.18	64.00	66.76	72.15	74.18
16	41.40	52.70	59.00	61.25	64.00	66.76	72.18	74.18
17	41.40	52.70	59.00	61.17	63.96	66.79	72.18	74.18
18	41.39	52.69	59.01	61.17	64.00	66.78	72.16	74.18
19	41.39	52.70	59.10	61.15	63.98	66.78	72.16	74.17
20	41.39	52.69	59.10	61.17	64.00	66.77	72.17	74.21

**Table 3:** Statistical analysis data of the values of fracture efforts (to the cut) of the number 3 Bard Parker scalpel blades from the Paramount® brand.

Normal statistics								
	1 Incision	2 Incision	3 Incision	4 Incision	5 Incision	6 Incision	7 Incision	8 Incision
Number of values	20	20	20	20	20	20	20	20
Minimum	47.9	51	51	52.2	58.9	65.2	60.01	71.29
25% Percentile	49.09	51.31	51.87	52.28	58.93	65.24	69	71.38
Median	49.6	51.38	51.9	52.29	58.96	65.24	69	71.38
75% Percentile	49.6	51.4	51.9	52.3	58.96	65.3	69.01	71.39
Maximum	49.88	51.41	52.1	52.3	58.96	65.36	69.15	71.4
Mean	49.38	51.33	51.84	52.29	58.94	65.26	68.56	71.38
Std. Deviation	0.4677	0.1167	0.2274	0.02231	0.02038	0.03881	2.014	0.02282
Std. Error	0.1046	0.02609	0.05085	0.004988	0.004558	0.008679	0.4503	0.005103
Lower 95% CI of mean	49.16	51.27	51.74	52.28	58.93	65.24	67.62	71.37
Upper 95% CI of mean	49.59	51.38	51.95	52.3	58.95	65.28	69.51	71.39
Sum	987.5	1027	1037	1046	1179	1305	1371	1428

ANOVA table								
Table analyzed	Data 1							
One-way analysis of variance								
P value	<0.0001							
Are means signif. different? (*P* < 0.05)	Yes							
Number of groups	8							
F	2793							
R square	0.9923							
Bartlett׳s statistic (corrected)	595.1							
*P* value	<0.0001							
Do the variances differ signif. (*P* < 0.05)	Yes							
ANOVA table	SS	df	MS					
Treatment (between columns)	10,611	7	1516					
Residual (within columns)	82.5	152	0.5427					
Total	10,694	159

**Table 4:** Statistical analysis data of the values of fracture efforts (to the cut) of the number 3 Bard Parker scalpel blades from the Elite® brand.

Normal statistics								
	1 Incision	2 Incision	3 Incision	4 Incision	5 Incision	6 Incision	7 Incision	8 Incision
Number of values	20	20	20	20	20	20	20	20
Minimum	41.3	52.6	58.91	61.15	63.96	66.6	72.13	74.1
25% Percentile	41.39	52.69	58.96	61.17	64	66.76	72.15	74.17
Median	41.39	52.69	59	61.18	64	66.78	72.15	74.18
75% Percentile	41.4	52.7	59	61.22	64.1	66.78	72.17	74.18
Maximum	41.41	52.7	59.1	61.29	64.18	66.8	72.18	74.22
Mean	41.39	52.69	58.99	61.19	64.03	66.76	72.15	74.18
Std. Deviation	0.02403	0.02207	0.05202	0.03407	0.05807	0.04464	0.01572	0.02236
Std. Error	0.005374	0.004935	0.01163	0.007619	0.01299	0.009981	0.003515	0.005
Lower 95% CI of mean	41.38	52.68	58.96	61.18	64.01	66.74	72.15	74.16
Upper 95% CI of mean	41.4	52.7	59.01	61.21	64.06	66.78	72.16	74.19
Sum	827.8	1054	1180	1224	1281	1335	1443	1484

ANOVA table								
Table analyzed	Data 1							
One-way analysis of variance								
*P* value	<0.0001							
Are means signif. different? (*P* < 0.05)	Yes							
Number of groups	8							
F	1,649,000							
R square	1							
Bartlett׳s statistic (corrected)	53.83							
*P* value	<0.0001							
Do the variances differ signif. (*P* < 0.05)	Yes							
ANOVA table	SS	df	MS					
Treatment (between columns)	15,936	7	2277					
Residual (within columns)	0.2098	152	0.001381					
Total	15,937	159						

## 2 Experimental design, materials and methods

### 2.1 Materials

In this data article, a number 3 Bard Parker scalpel blades from the Paramount® and Elite® brands, both acquired from commercial houses existent in the market.

### 2.2 Experimentation

As the experimentation model, commercially available ex vivo pig mandibles were used. The scalpel blades were taken to the Shimadzu EZ-S Texture Analyzer with a maximum capacity of 500 N of pressure for the performance of the penetrating mucoperiosteal cuts with a 45° angle. The penetrating incision was performed at a constant speed of 10 mm per minute until the bone structure was reached. The photographs were standardized by using a form, where it was positioned in a unified form and avoiding movements from the scalpel blade (Fig. 1).

### 2.3 Statistical analysis

The significance of means within the groups of experimental data was evaluated using one-way analysis of variance (one-way ANOVA).
